# UXT antisense RNA 1 sever as a novel prognostic long non-coding RNA in early stage pancreatic ductal adenocarcinoma patients after receiving pancreaticoduodenectomy

**DOI:** 10.7150/jca.46084

**Published:** 2021-02-21

**Authors:** Xiwen Liao, Rui Huang, Xiangkun Wang, Ketuan Huang, Chengkun Yang, Xin Zhou, Chuangye Han, Hao Su, Xinping Ye, Kang Liu, Guangzhi Zhu, Tao Peng

**Affiliations:** 1 Department of Hepatobiliary Surgery, The First Affiliated Hospital of Guangxi Medical University, Nanning, 530021, Guangxi Zhuang Autonomous Region, People's Republic of China.; 2 Department of Hematology, The First Affiliated Hospital of Guangxi Medical University, Nanning, 530021, Guangxi Zhuang Autonomous Region, People's Republic of China.; 3 Department of Radiation Oncology, The First Affiliated Hospital of Guangxi Medical University, Nanning 530021, Guangxi Zhuang Autonomous Region, People's Republic of China.

**Keywords:** UXT antisense RNA 1, The Cancer Genome Atlas, pancreatic ductal adenocarcinoma, pancreaticoduodenectomy, prognosis

## Abstract

**Objective:** The principal objective of this project was to investigate the prognostic value of UXT antisense RNA 1 (UXT-AS1) in pancreatic ductal adenocarcinoma (PDAC), as well as its biological function mechanisms and the screening of targeted drugs using The Cancer Genome Atlas (TCGA) PDAC genome-wide RNA sequencing (RNA-seq) dataset.

**Methods:** We used TCGA 112 early stage PDAC patients to screen the prognostic value of UXT-AS1. Biological functions and mechanisms of UXT-AS1 were investigated by co-expression analysis, differentially expressed genes (DEGs) and gene set enrichment analysis, while targeted drug screening was investigated by connectivity Map (CMap).

**Results:** By analyzing the dataset from TCGA cohort, we found that UXT-AS1 was significantly up-regulated in pancreatic cancer tissues. Multivariate survival analysis demonstrated that PDAC patients with high UXT-AS1 expression had an unfavourable prognosis (adjusted P=0.033, HR=1.830, 95%CI=1.051-3.188). Genome-wide co-expression analysis and gene set enrichment analysis suggested that UXT-AS1 may act as a pivotal part in PDAC by participating in nuclear factor kappa beta, regulation of tumor necrosis factor, cell adhesion, T cell receptor signaling pathway, and numerous immune-related biological processes and signaling pathways. Functional enrichment analysis of DEGs between high- and low-UXT-AS1 expression groups suggested that these DEGs were significant enriched in B cell receptor complex, response to drug chemical carcinogenesis and drug metabolism-cytochrome P450. CMap analysis revealed that quipazine and terazosin may be targeted drugs for UXT-AS1 in PDAC.

**Conclusion:** Our current study has identified UXT-AS1 as a novel biomarker for the prognosis of early stage PDAC. We also clarified its biological functional mechanisms and identified two targeted drugs of UXT-AS1 in PDAC.

## Introduction

Pancreatic cancer (PC) is a digestive tract cancer that has a highly malignant and is difficult to diagnose and treat 1. About ninety percent of them are pancreatic ductal adenocarcinoma (PDAC) that originate from the epithelial ducts. The morbidity and mortality recently increased significantly 2. The five years survival rate is lower than most cancers, which is one of the worst prognostic malignancies 3. The early diagnosis of PC is not high, the surgical mortality is high, and the cure rate is low. Therefore, there is an urgent need to develop biomarkers with higher specificity and sensitivity in PC.

A growing number of evidence demonstrated that long non-coding RNA (lncRNA) plays a crucial function in tumorigenesis, cancer progression and prognosis 4, 5. Previous study have shown that UXT antisense RNA 1 (UXT-AS1) is significantly up-regulated in colorectal cancer (CRC) tumor tissues, while the clinical outcome of CRC patients with high UXT-AS1expression is unfavourable 6. *In vitro* cytology experiments indicate that UXT-AS1 functions as an oncogene in CRC 6. Through literature search, until now, there are no other studies reveal the clinical significance and functional mechanism of UXT-AS1 in cancers. Our previous study used The Cancer Genome Atlas (TCGA) PDAC cohort to screen a series of prognostic related biomarkers for PDAC 7, 8. In order to further understand the clinical significance and functional mechanisms of UXT-AS1 in cancers. The principal objective of this project was to investigate the prognostic value of UXT-AS1 in PDAC, as well as its biological function mechanisms and the screening of targeted drugs using TCGA PDAC genome-wide RNA sequencing (RNA-seq) data set.

## Materials and methods

### Data collation

**Figure 1:**
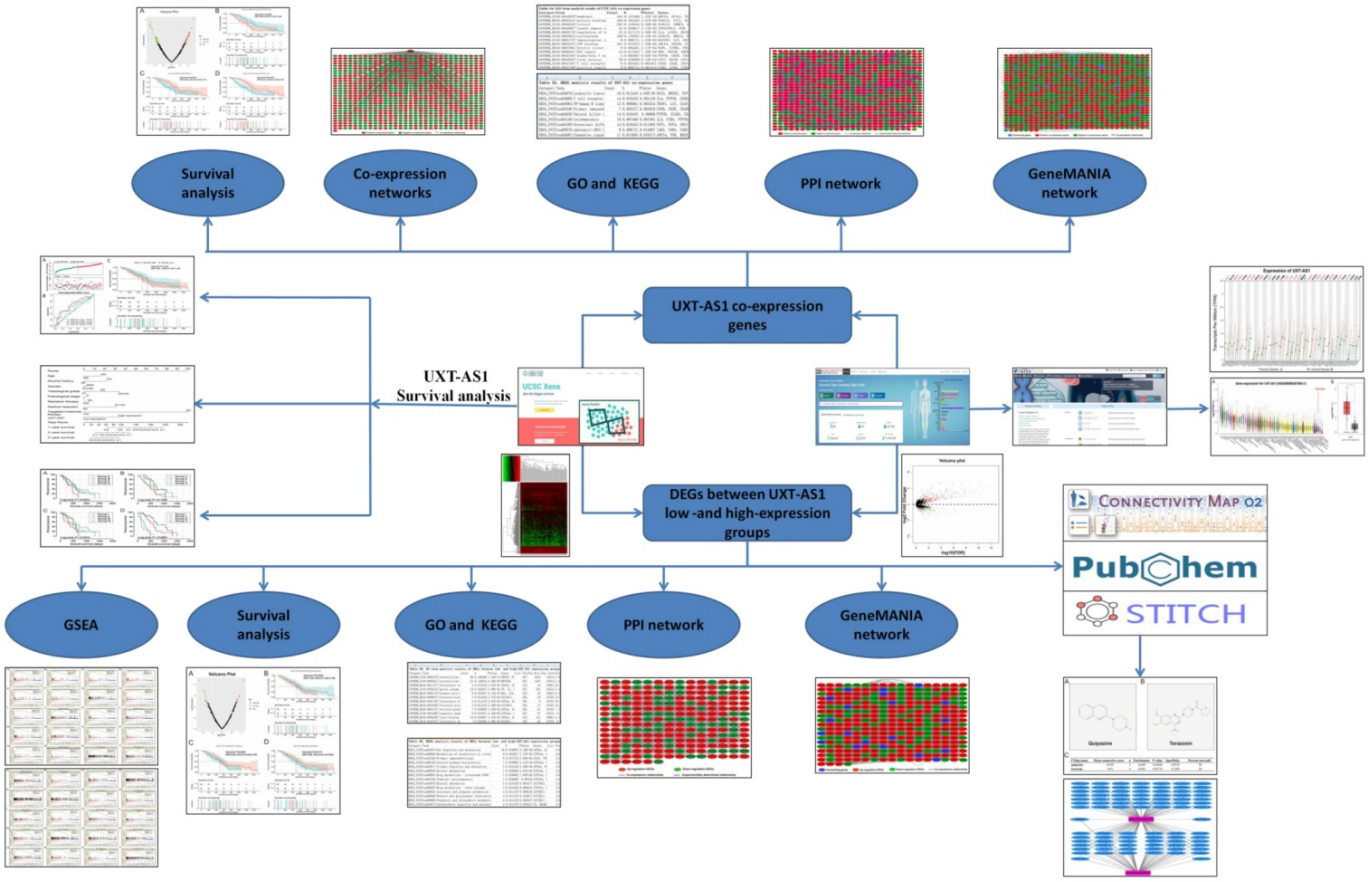
Flow chart of current study.

The flow chart of our study is summarized in **Figure 1**. We obtained the PDAC RNA-seq data set from the TCGA website (https://portal.gdc.cancer.gov) and the corresponding clinical parameters and prognosis information from the University of California, Santa Cruz (UCSC) Xena (http://xena.ucsc.edu) 9. Raw RNA-seq data set normalization is performed using the DESeq package 10. We also obtained the distribution diagram of UXT-AS1 expression in normal human organs tissues from the GTEx Portal (http://www.gtexportal.org/home/) 11, 12, as well as the scatter plot of the UXT-AS1expression profile between tumor and normal tissues in TCGA pan-cancer cohort from GEPIA (http://gepia.cancer-pku.cn/index.html) 13. Inclusion criteria and exclusion criteria for TCGA PDAC cohort patients included in the further prognostic analysis are detailed in our previously published studies 7, 8. There were 112 patients with early PDAC, who receiving pancreaticoduodenectomy, were fit into the subsequent survival analysis. All the datasets in current study were got from open access databases and do not involve any animal or human study. Therefore, no additional ethics committee approval is need.

### Prognostic value of UXT-AS1 in early stage PDAC

Multivariate Cox proportional risk regression model was used to evaluate the prognostic value of UXT-AS1 in PDAC, and combined survival analysis to evaluate the predictive value of UXT-AS1 in combination with clinical parameters. In addition, UXT-AS1 and clinical parameters were also used to construct the prognostic nomogram model by using the “rms” package in R version 3.6.2 software.

### Function Exploration of UXT-AS1 in PDAC

We all know that lncRNA functions by regulating its downstream target protein coding genes (PCGs). For biological function mechanisms of UXT-AS1 in PDAC, in this study, we used the whole-genome RNA-seq dataset to screen its co-expression genes in PDAC tumor tissues, and then using the co-expression genes to explore the biological function mechanisms by the Database for Annotation, Visualization and Integrated Discovery (DAVID) v6.8 (https://david.ncifcrf.gov/home.jsp). The co-expression relationship of UXT-AS1 co-expression genes were also verify by GeneMANIA (http://genemania.org/) and STRING (https://string-db.org) online tools. In addition, edgeR was used to screen differentially expressed genes (DEGs) between high-and low-UXT-AS1 expression groups, and the above bioinformatics enrichment method was used to carry out functional enrichment of these DEGs 14. We also use gene set enrichment analysis (GSEA: http://software.broadinstitute.org/gsea/index.jsp) to further explore the mechanisms between high-and low-UXT-AS1 expression groups 15, 16. The prediction of targeted drugs was conducted on the Connectivity Map (CMap: https://portals.broadinstitute.org/cmap/)online tool by DEG between low- and high-UXT-AS1 groups 17. DEG's gene symbol are converted to an affymetrix probe set ID, then import the up- and down-regulated DEG gene lists into the CMAP website for quick query. The targeted drug and gene interaction networks were analyzed by STITCH online tools 18, 19, while the chemical structure of drugs were analyzed by Pubchem (https://pubchem.ncbi.nlm.nih.gov).

### Statistical analysis

The screening of UXT-AS1 co-expression PCGs depends on Pearson correlation coefficient. DEGs identified by edgeR were set as: |log_2_ fold change (FC)|>1, P<0.05 and false discovery rate (FDR) <0.05. GSEA results were set as: |Normalized Enrichment Score (NES)|>1, P<0.05 and FDR<0.25. Log-rank test and Cox proportional risk regression model were selected for survival analysis. P<0.05 was considered statistically significant. The statistical software involved in this study is R version 3.6.2 and SPSS version 22.0.

## Results

### Expression distribution and prognostic value evaluation of UXT-AS1

**Figure 2:**
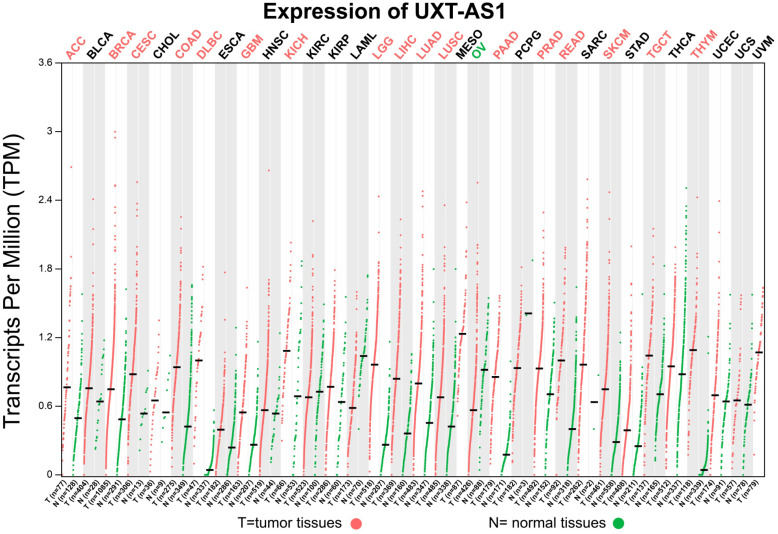
Scatter plot of UXT-AS1 expression between tumor and corresponding normal tissues of TCGA pan-cancer cohort. **Abbreviation**: PAAD: pancreatic adenocarcinoma.

**Figure 3:**
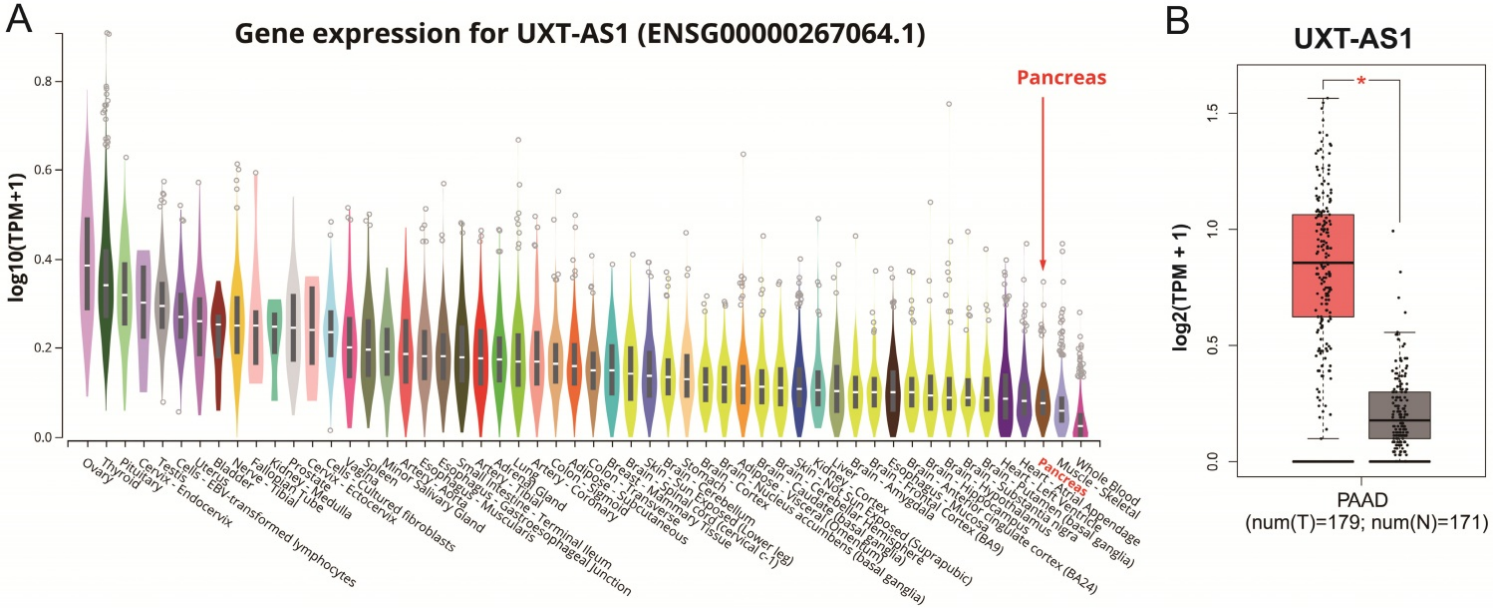
The distribution of UXT-AS1 in normal organ tissues and pancreatic cancer tissues. (A): Violin plot of UXT-AS1 expression distribution in normal organ tissues; (B): Box plot of UXT-AS1 expression distribution between normal pancreas and pancreatic adenocarcinoma tissues. Note: * represent P<0.05.

**Figure 4:**
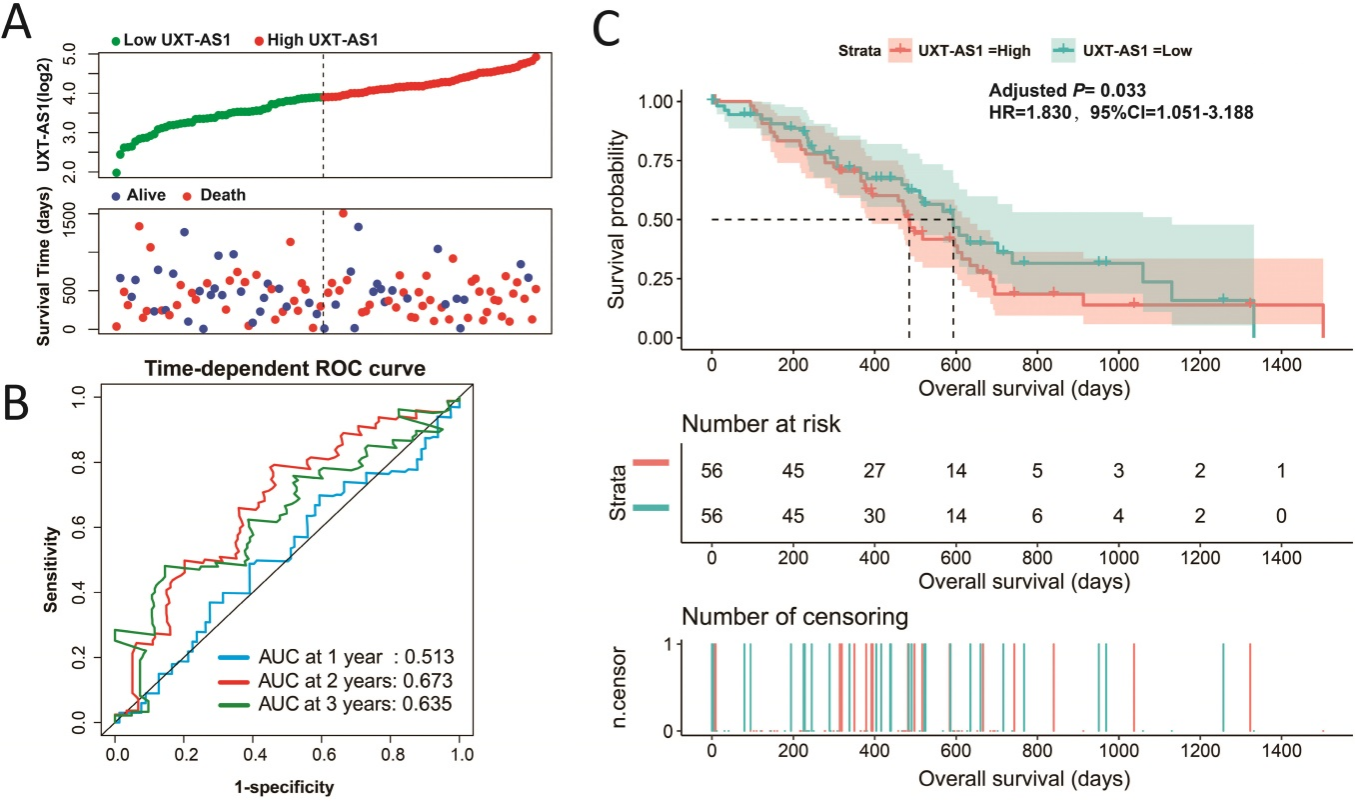
Evaluation of UXT-AS1 prognostic value in PDAC. (A): Distribution scatter plot of survival time and UXT-AS1 expression level; (B): Time-dependent ROC curve of UXT-AS1 predicting PDAC survival; (C): Kaplan-Meier survival curve between high- and low-UXT-AS1 expression groups.

**Figure 5:**
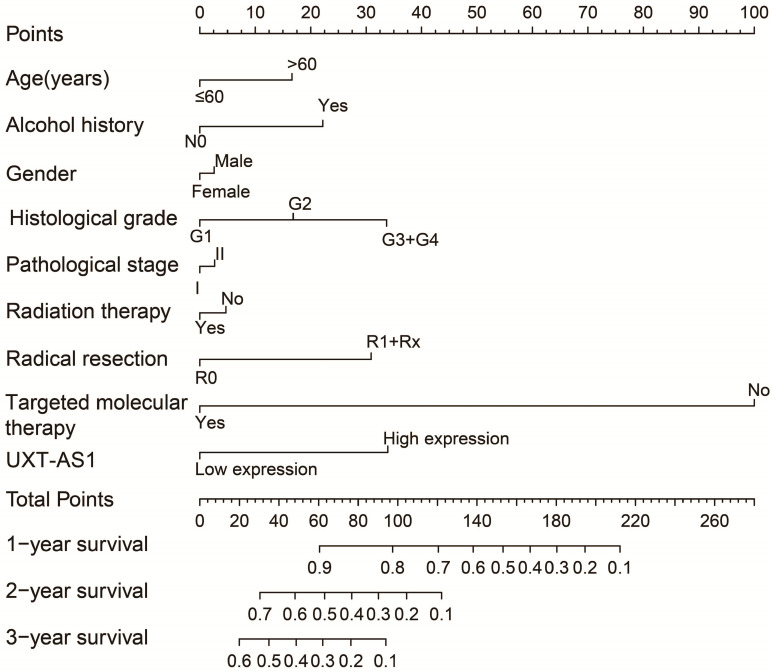
A nomogram model constructed from the PDAC clinical parameters and UXT-AS1 expression.

**Figure 6:**
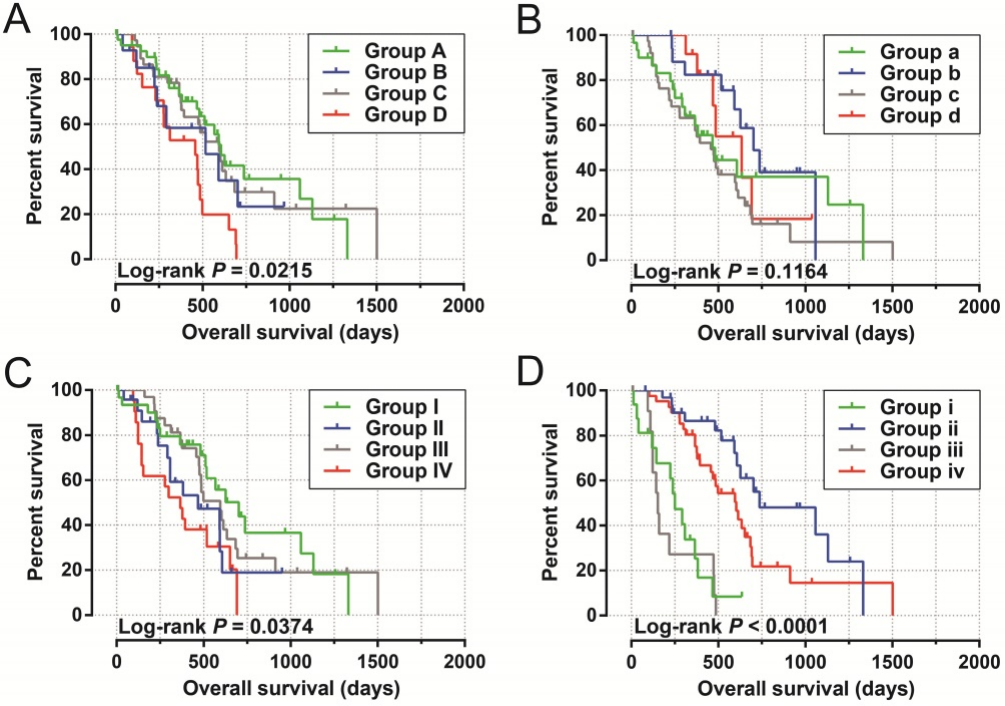
Joint effect survival analysis by PDAC clinical parameters and UXT-AS1 expression. (A): Histological Grade and UXT-AS1 combination; (B): Radiation therapy and UXT-AS1 combination; (C): Radical resection and UXT-AS1 combination; (D): Targeted molecular therapy and UXT-AS1 combination.

By analyzing the distribution of expression levels in tumor and corresponding normal tissues in the TCGA pan-cancer cohort, we found that UXT-AS1 was markedly up-regulated in most of cancers, but only down-regulated in ovarian cancer (**Figure 2**). By comparing the expression distribution of UXT in normal human organ tissues, we found that UXT-AS1 was low expression in normal pancreatic tissues (**Figure 3A**). By analyzing the expression distribution of patients with PC in TCGA cohort, we found that UXT-AS1 was markedly up-regulated in PC tissues (**Figure 3B**). A total of 112 PDAC patients were fit into prognostic analysis, and the clinical data indicated that histological grade, radical resection, radiation therapy and targeted molecular therapy were markedly correlated with the prognosis of PDAC (**Table S1**). The above four factors should be included in the multivariate Cox proportional risk regression model for correction. Survival analysis suggest that high-UXT-AS1 expression of PDAC was related to an unfavourable prognosis (**Figure 4A-C**, log-rank P=0.266, adjusted P=0.033, HR=1.830, 95%CI=1.051-3.188). We used survivalROC to evaluate the accuracy of prognosis prediction and found that UXT-AS1 had the highest efficiency in predicting two-year overall survival of PDAC, which was 0.673 (**Figure 4B**). We then constructed a nomogram model containing UXT-AS1 and clinical parameters of PDAC to predict individual patient outcomes. We found that the contribution of UXT-AS1 to prognosis in our PDAC cohort was second only to the targeted molecular therapy (**Figure 5**). Joint effect survival suggest that high UXT-AS1 expression combine with grade G3/G4 PDAC patients were significantly increased risk of death, by comparing with low UXT-AS1 expression combine with grade G1/G2 groups (adjusted P=0.0005, HR=3.592, 95%CI=1.749-7.375,**Table S2** and** Figure 6A**). In patients did not receiving radiation therapy subgroups, we also observed that high UXT-AS1 PDAC patients have an unfavourable prognosis by comparing with low UXT-AS1 expression patients (adjusted P=0.02, HR=2.166, 95%CI=1.127-4.164,**Table S2** and** Figure 6B**). High UXT-AS1 expression PDAC patients without radical resection were significantly increased risk of death, by comparing with radical resection PDAC patients with low UXT-AS1 expression groups (adjusted P=0.002, HR=3.563, 95%CI=1.583-8.019,**Table S2** and** Figure 6C**). Both the PDAC patients with low (adjusted P<0.0001, HR= 0.128, 95%CI=0.048-0.341,**Table S2** and** Figure 6D**) or high (adjusted P=0.0004, HR=0.242, 95%CI=0.110-0.529,**Table S2** and** Figure 6D**) UXT-AS1 expression combine with targeted molecular therapy were significantly reduced risk of death, by comparing with these did not receiving targeted molecular therapy combine with low UXT-AS1 expression groups patients.

### Functional enrichment of UXT-AS1 using whole-genome co-expression analysis

**Figure 7:**
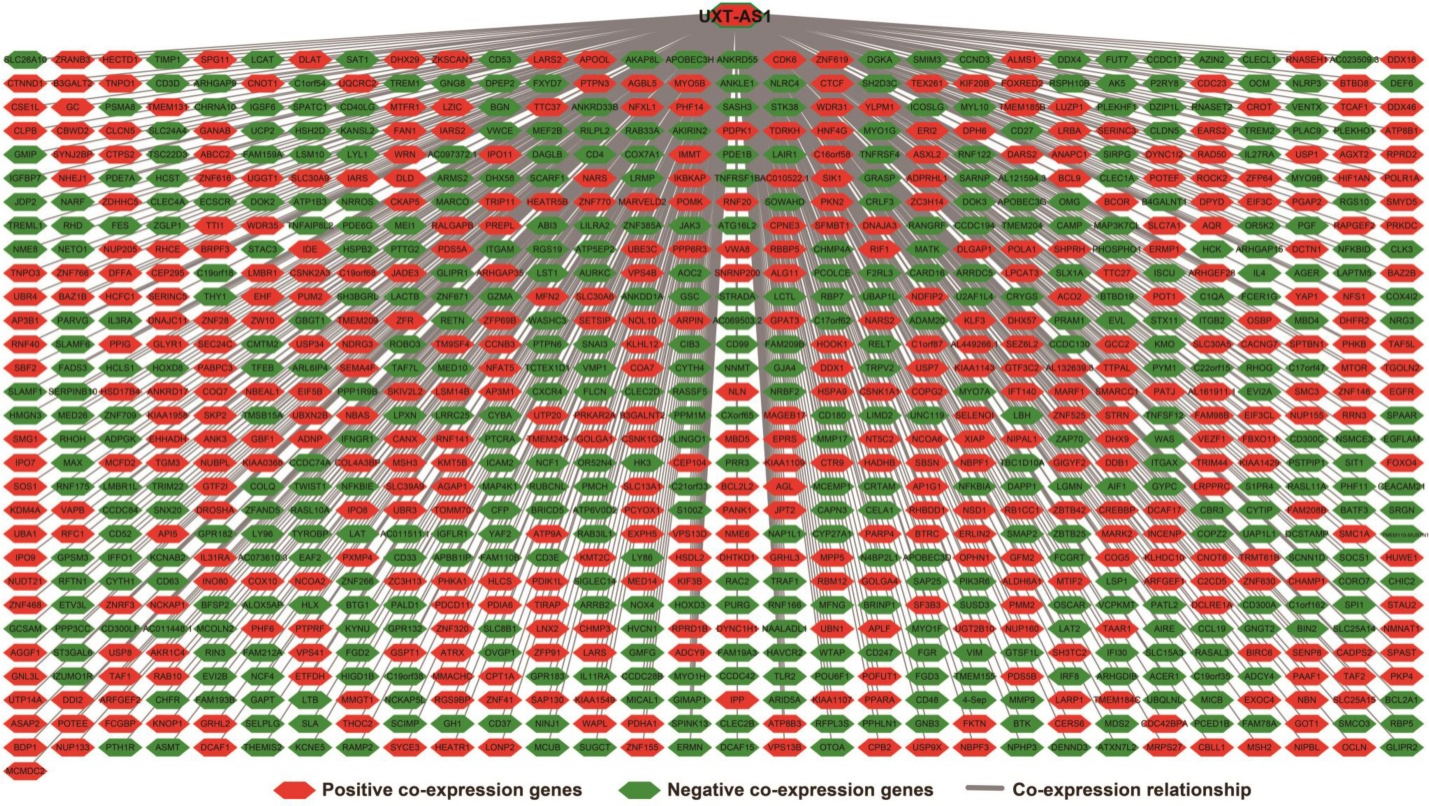
Co-expression network of UXT-AS1 and its co-expression PCGs in PDAC tumor tissues.

**Figure 8:**
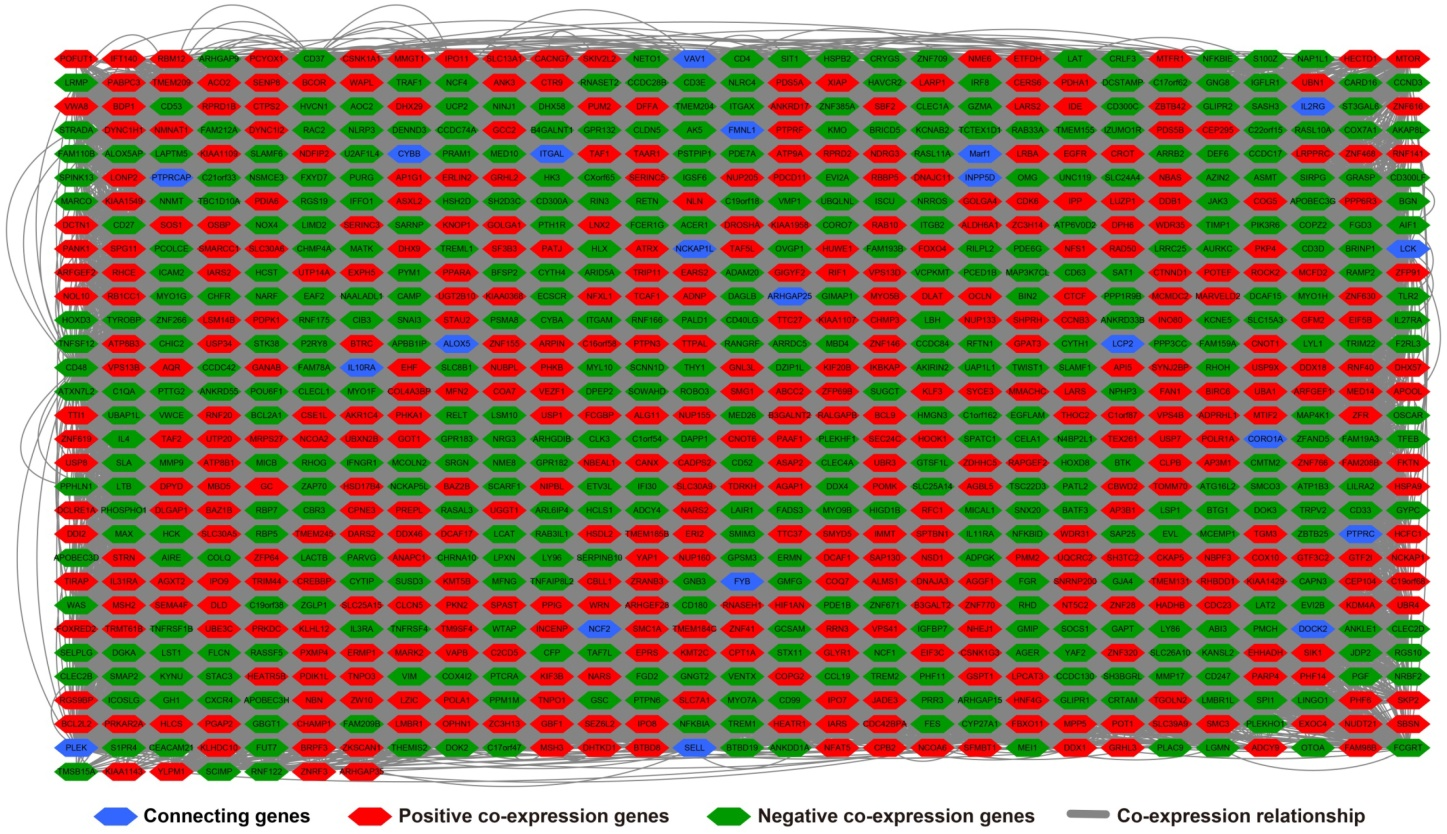
Co-expression network of UXT-AS1 co-expression PCGs constructed by GeneMANIA.

**Figure 9:**
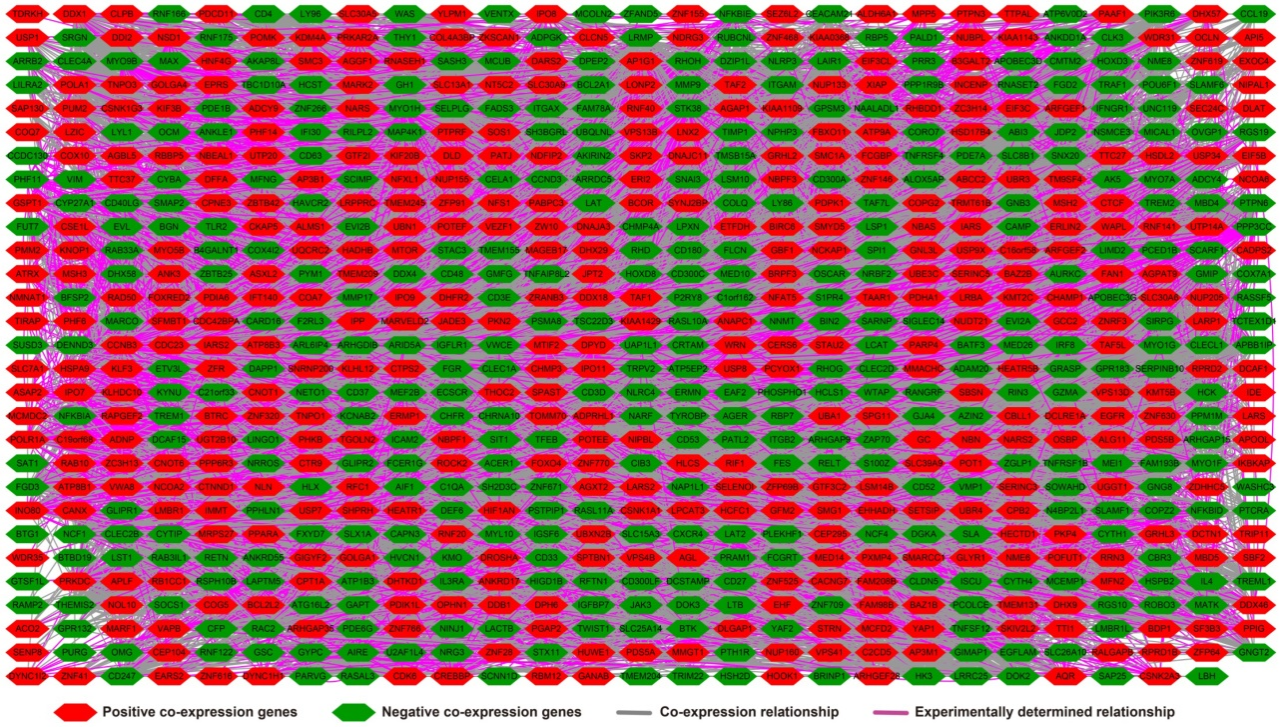
Gene-gene interaction regulatory network of UXT-AS1 co-expression PCGs constructed by STRING.

**Figure 10:**
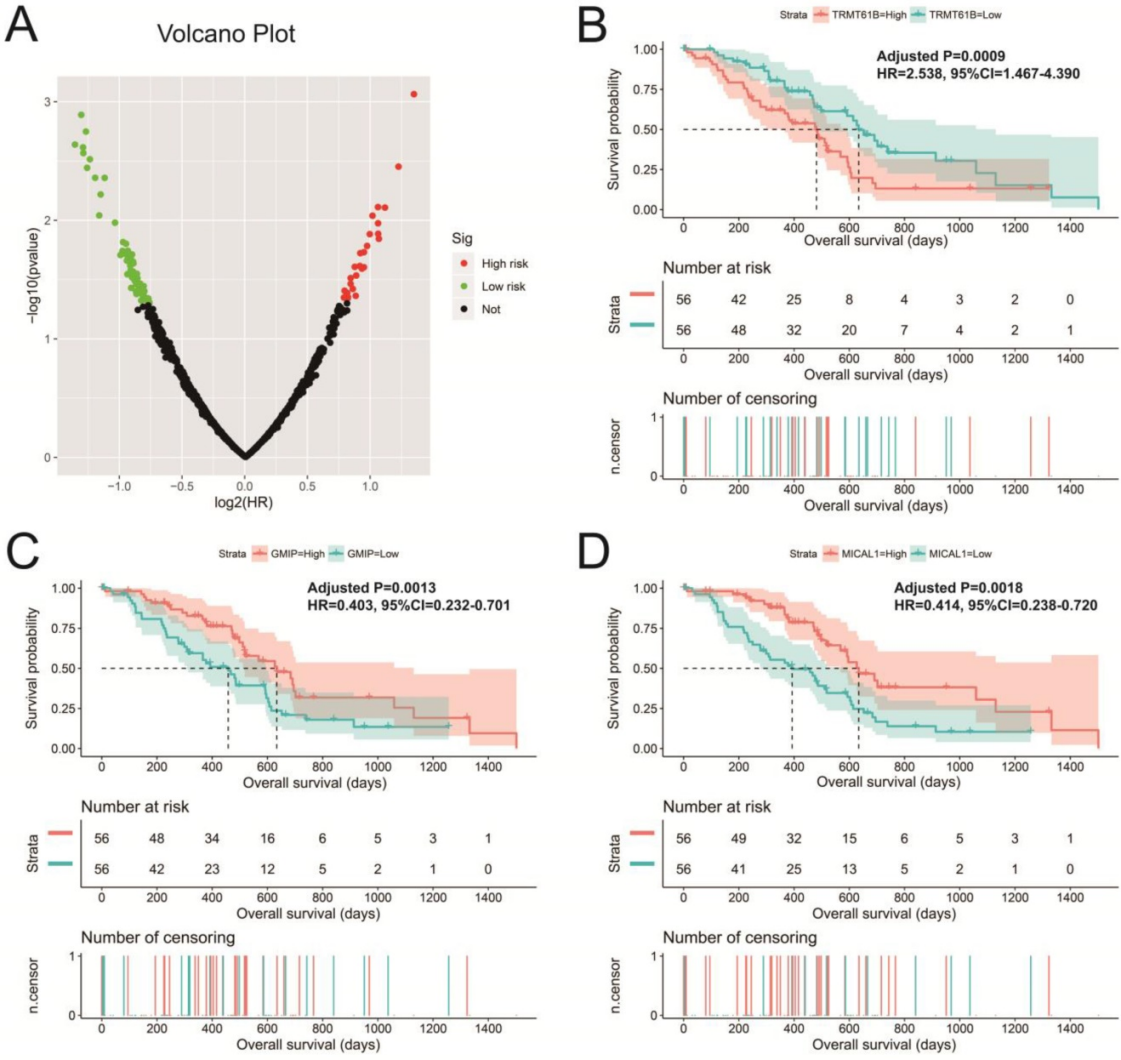
Survival analysis of UXT-AS1 co-expression PCGs in PDAC. (A): Volcano plot of survival analysis results of UXT-AS1 co-expression PCGs; (B): Kaplan-Meier survival curve of TRMT61B; (C): Kaplan-Meier survival curve of GMIP; (D): Kaplan-Meier survival curve of MICAL1.

There are 901 genes were determined as UXT-AS1 co-expression PCGs, of them, 456 were negative co-expression PCGs and 445 positive co-expression PCGs (**Table S3 and Figure 7**). Furthermore, we also used GeneMANIA (**Figure 8**) and STRING (**Figure 9**) to analyze the gene-gene interaction of these co-expressed PCGs, and we could observe a complex co-expression interaction regulation relationship networks among these PCGs, indicating that these PCGs are related to each other through UXT-AS1, and then play a role in PDAC. Functional enrichment analysis of gene ontology (GO) term reveal that UXT-AS1 co-expressed PCGs markedly refer to the regulation of the following biological processes: regulation of immune response, mitotic sister chromatid cohesion, DNA repair, positive regulation of tumor necrosis factor production, positive regulation of T cell proliferation, positive regulation of interleukin-12 production, toll-like receptor 4 signaling pathway, regulation of ARF protein signal transduction, regulation of cell proliferation, positive regulation of interleukin-4 production, stem cell population maintenance, cell surface receptor signaling pathway, positive regulation of interleukin-10 production, T cell receptor signaling pathway, positive regulation of NF-kappaB transcription factor activity, and regulation of cell adhesion (**Table S4**). Kyoto Encyclopedia of Genes and Genomes (KEGG) suggest that UXT-AS1 co-expressed PCGs were significantly enriched in leukocyte transendothelial migration, primary immunodeficiency, T cell receptor signaling pathway, NF-kappa B signaling pathway, natural killer cell mediated cytotoxicity, and chemokine signaling pathway (**Table S5**). In addition, we also performed a multivariate survival analysis of these UXT-AS1 co-expressed PCGs. A total of 91 PDAC prognostic UXT-AS1 co-expressed PCGs were obtained, of which 28 were PDAC high risk genes (HR>1) and 63 were low risk (HR<1) (**Figure 10A** and **Table S6** ). The top three genes with a minimum P values were TRNA methyltransferase 61B (TRMT61B, adjusted P=0.0009, HR=2.538, 95%CI=1.467-4.390,** Figure 10B**), GEM interacting protein (GMIP, adjusted P=0.0013, HR=0.403, 95%CI=0.232-0.701,** Figure 10C**)and microtubule associated monooxygenase, calponin and LIM domain containing 1 (MICAL1, adjusted P=0.0018, HR=0.414, 95%CI=0.238-0.720,** Figure 10D**).

### Biological function differences between low- and high-UXT-AS1 phenotypes

**Figure 11:**
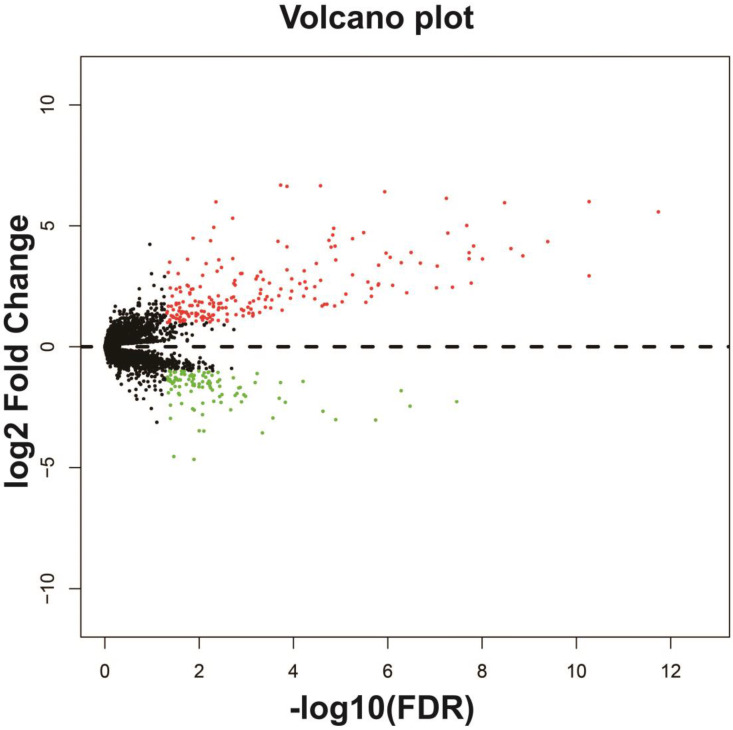
Volcano plot of differentially expressed genes between high- and low-UXT-AS1 expression groups. Notes: red dots represent up-regulated genes; green dots represent down-regulated genes.

**Figure 12:**
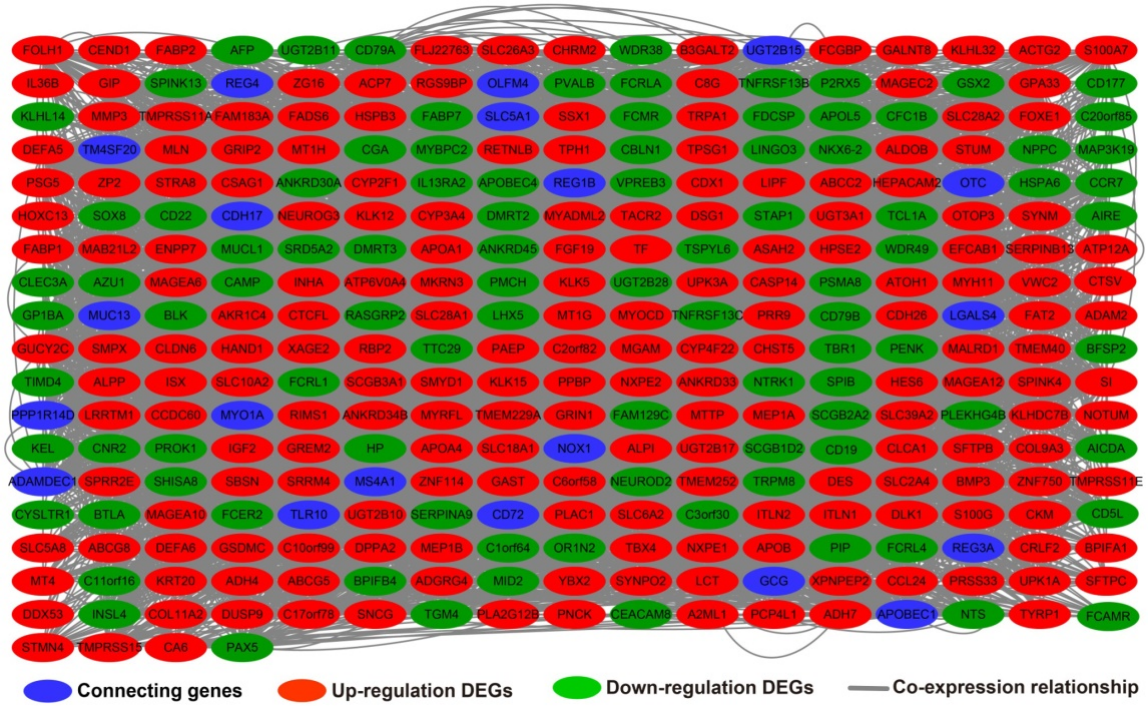
Co-expression network of DEGs constructed by GeneMANIA.

**Figure 13:**
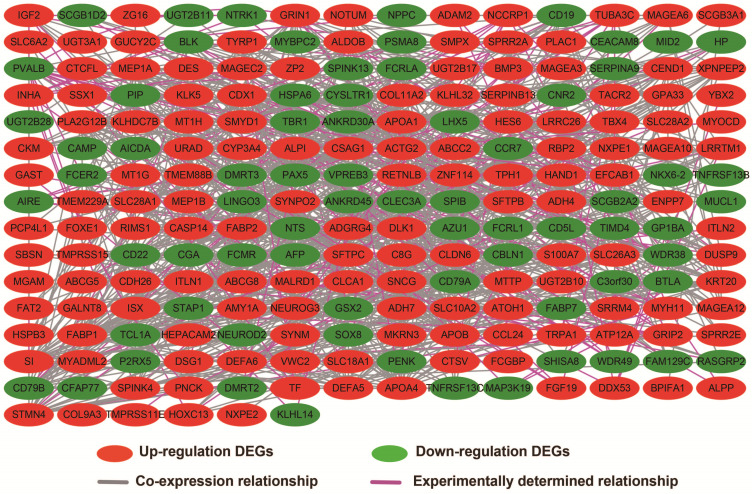
Gene-gene interaction regulatory network of DEGs constructed by STRING.

**Figure 14:**
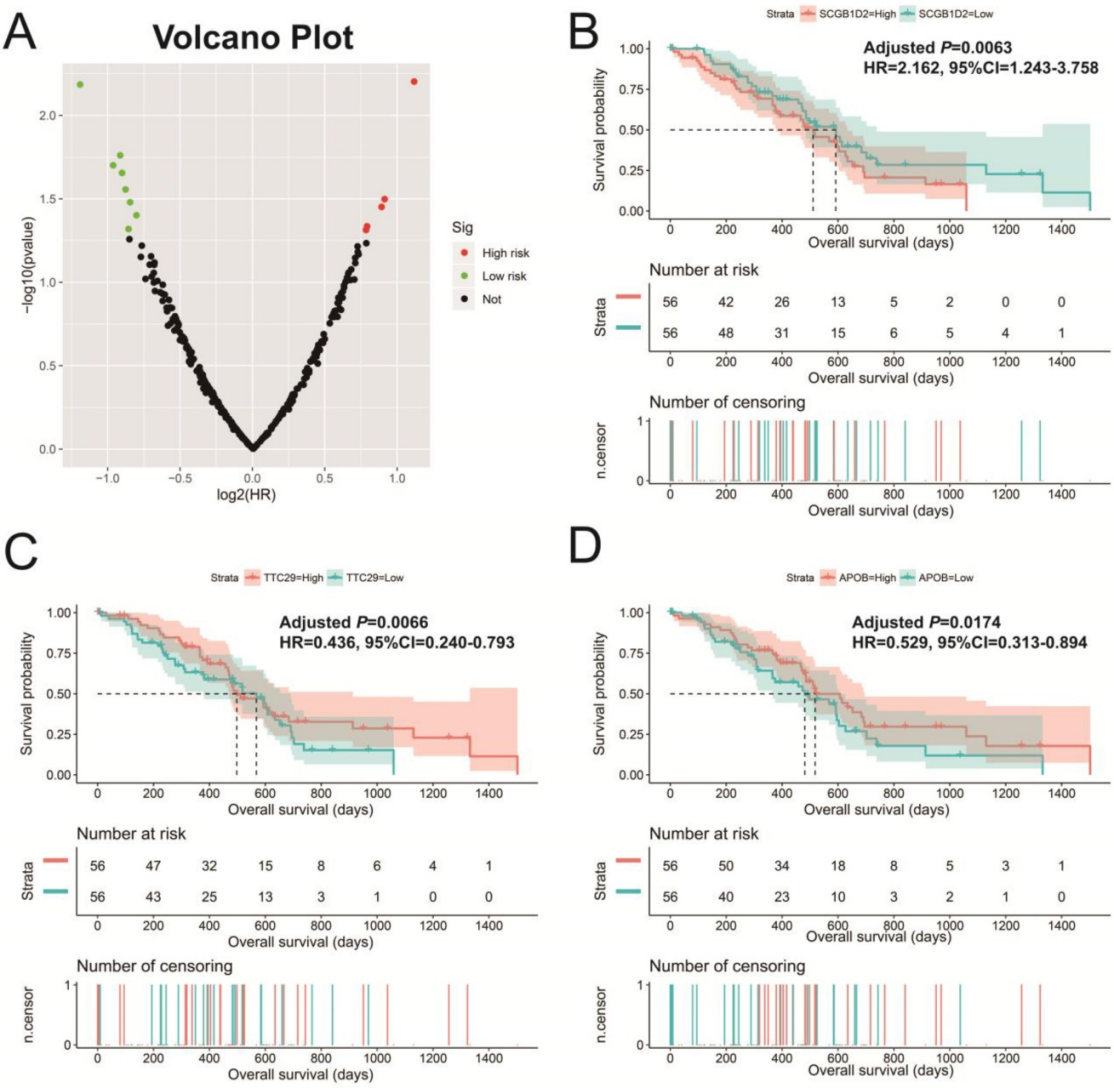
Survival analysis of DEGs in PDAC. (A): Volcano plot of survival analysis results of DEGs; (B): Kaplan-Meier survival curve of SCGB1D2; (C): Kaplan-Meier survival curve of TTC29; (D): Kaplan-Meier survival curve ofAPOB.

In addition to exploring the functions of UXT-AS1 in PDAC from co-expressed PCGs, edgeR was used to identify differentially expressed genes (DEGs) between high- and low-UXT-AS1 expression groups, and further explored the mechanisms of UXT-AS1 through DEGs function enrichment analysis. There were 313 DEGs identified by edgeR, of which 102 DEGs were down-regulation and 211 DEGs were up-regulation (**Table S7** and **Figure 11**). Gene-gene interaction analysis by GeneMANIA (**Figure 12**) and STRING (**Figure 13**) suggest that these DEGs have intricate co-expression interactions and experimentally determined regulatory relationship networks. GO term analysis suggest that these DEGs notably enriched in retinoid metabolic process, glucuronosyltransferase activity, toxic substance binding, response to drug, adaptive immune response, antioxidant activity and B cell receptor complex (**Table S8**). KEGG enrichment suggest that these DEGs were notably related to metabolism of xenobiotics by cytochrome P450, primary immunodeficiency, drug metabolism - cytochrome P450, chemical carcinogenesis and drug metabolism - other enzymes (**Table S9**). Multivariate survival analysis suggest that there were 13 DEGs were identified as prognostic DEGs, of which eight were PDAC high risk genes and five were low risk (**Table S10** and** Figure 14A**). The top three genes with a minimum P values were secretoglobin family 1D member 2 (SCGB1D2, adjusted P=0.0063, HR=2.162, 95%CI=1.243-3.758,** Figure 14B**), tetratricopeptide repeat domain 29 (TTC29 adjusted P=0.0066, HR=0.436, 95%CI=0.240-0.793,** Figure 14C**) and apolipoprotein B (APOB, adjusted P=0.0174, HR=0.529, 95%CI=0.313-0.894,** Figure 14D**).

**Figure 15:**
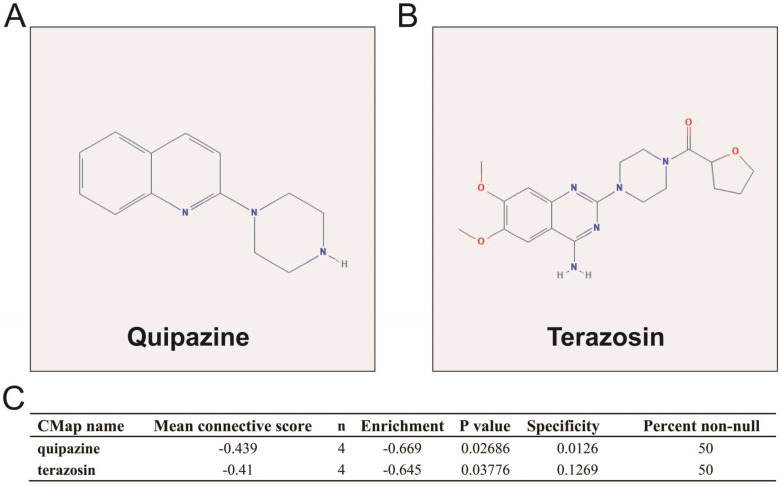
CMap analysis results of UXT-AS1. (A): The compound structure of quipazine; (B): The compound structure of terazosin; (C): CMap analysis result.

**Figure 16:**
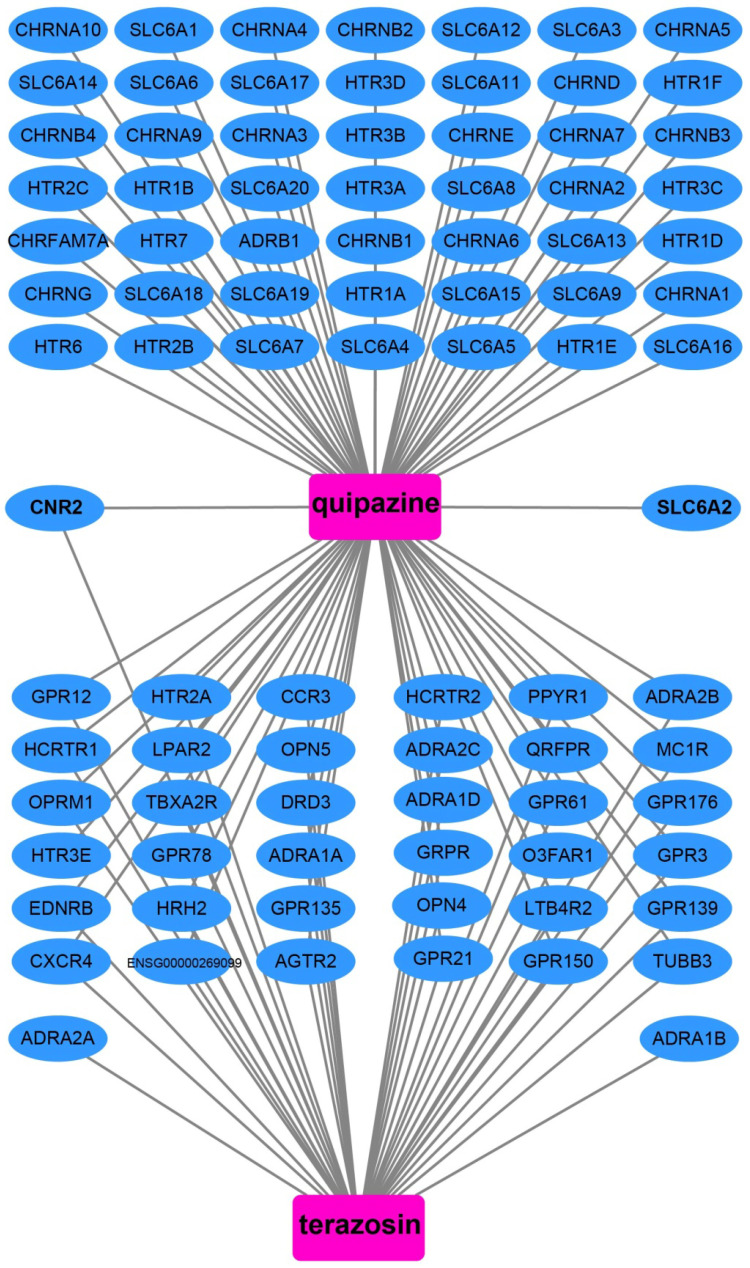
Drug-gene interaction regulatory network of quipazine and terazosin, which constructed by STITCH.

We also used DEGs between high and low UXT-AS1 expression groups to screen UXT-AS1 targeted drugs on CMap online tool. Through CMap analysis, we screened 2 compounds that may be targeted therapy drugs of UXT-AS1 in PDAC. These two drugs are quipazine (mean connective score=-0.439, P=0.02686, **Figure 15A and C**) and terazosin (mean connective score=-0.41, P=0.03776, **Figure 15B-C**). Subsequently, we also used the STITCH online tool to construct the drug-gene interaction regulatory networks for the two drugs (**Figure 16**). In the drug-gene interaction networks, we found that the two drugs had a great many of overlapping interaction genes, and we also found that two of these genes were DEGs between low- and high-UXT-AS1 expression groups. The two DEGs were cannabinoid receptor 2 (CNR2: down-regulated) and solute carrier family 6 member 2 (SLC6A2: up-regulated). Based on this, we speculate that quipazine and terazosin may play a targeted drug role in PDAC by regulating CNR2, while quipazine may also play a part through SLC6A2 in PDAC.

**Figure 17:**
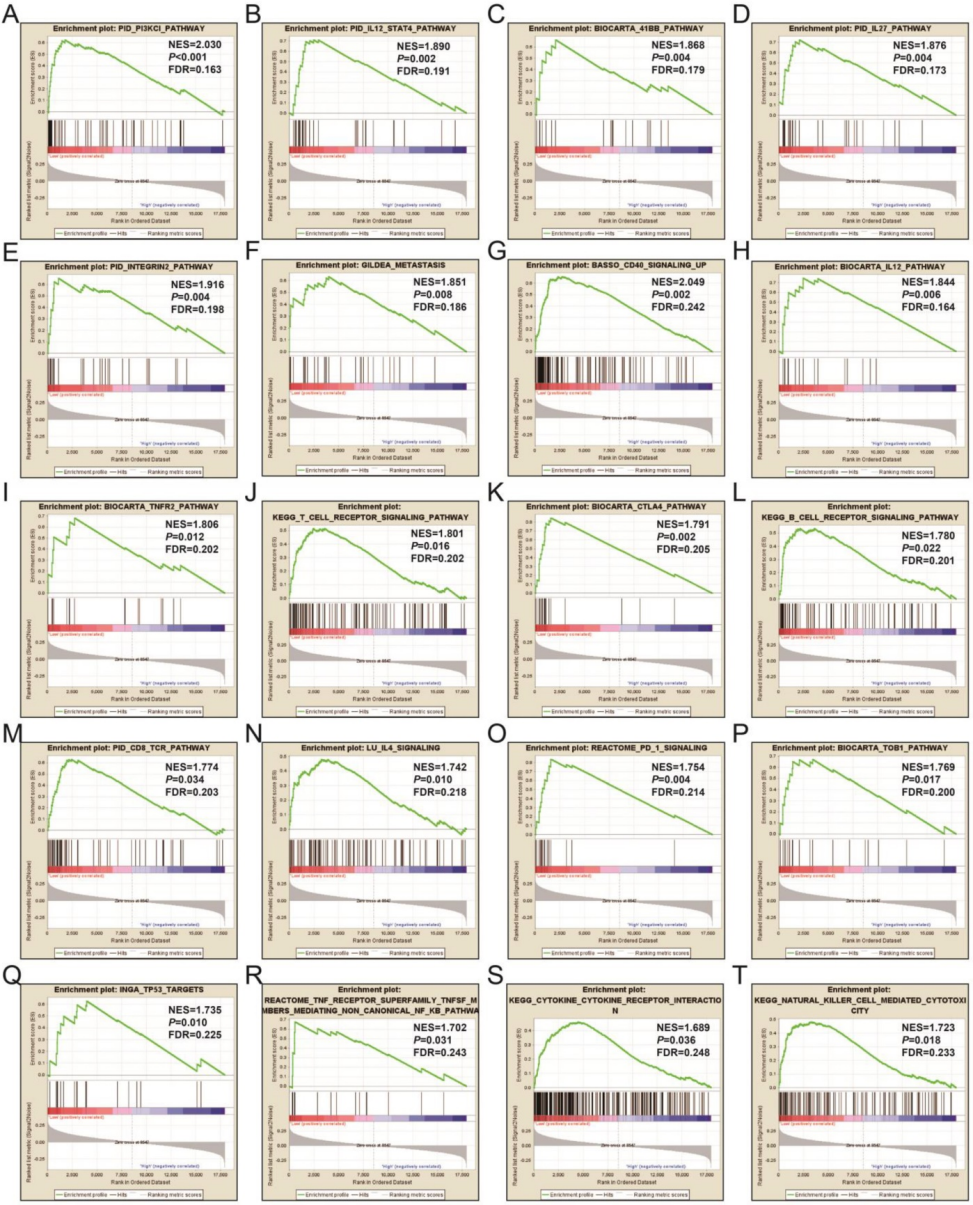
GSEA results of UXT-AS1 in PDAC using the c2 reference gene set. (A): PID PI3KCI PATHWAY; (B): PID IL12 STAT4 PATHWAY; (C): BIOCARTA 41BB PATHWAY; (D): OID IL27 PATHWAY; (E): PID INTEGRUB2 PATHWAY; (F): GILDEA METASTASIS; (G): BASSO CD40 SIGNALING UP; (H): BIOCARTA IL12 PATHWAY; (I): BIOCARTA TNFR2 PATHWAY; (J): KEGG T CELL RECEPTOR SIGNALING PATHWAY; (K): BIOCARTA CTLA4 PATHWAY; (L): KEGG B CELL RECEPTOR SIGNALING PATHWAY; (M): PID CD8 TCR PATHWAY; (N): LU IL4 SIGNALING; (O): REACTOME PD 1 SIGNALING; (P): BIOCARTA TOB1 PATHWAY; (Q): INGA TP53 TARGETS; (R): REACTOME TNF RECEPTOR SUPERFAMILY TNFSF MEMBER MEDIATING NON CANONICAL NF KB PATHWAY; (S): KEGG CYTOKINE RECEPTOR INTERACTION; (T): KEGG NATURAL KILLER CELL MEDIATED CYTOTOXICITY. Notes: FDR, false discovery rate; NES: normalized enrichment score; ES: enrichment score.

**Figure 18:**
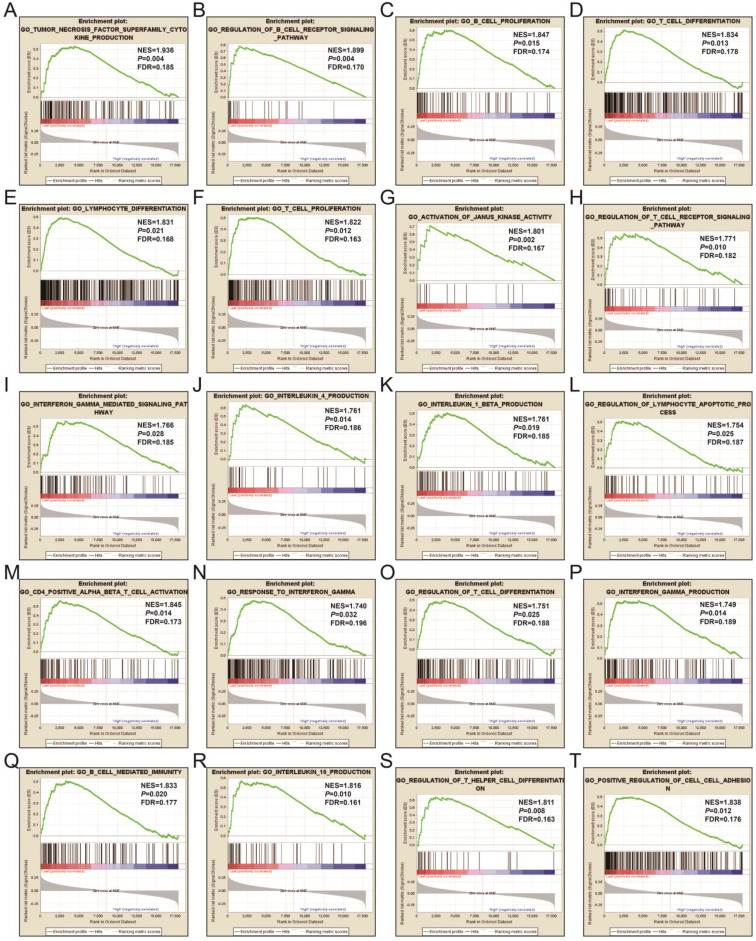
GSEA results of UXT-AS1 in PDAC using the c5 reference gene set. (A): GO TUMOR NECROSIS FACTOR SUPERFAMILY CYTOKINE PRODUCTION; (B): GO REGULATION OF B CELL RECEPTOR SIGNALING PATHWAY; (C): GO B CELL PROLIFERATION; (D): GO T CELL DIFFERENTIATION; (E): GO LYMPHOCYTE DIFFERENTIATION; (F): GO T CELL PROLIFERATION; (G): GO ACTIVATION OF JANUS KINASE ACTIVITY; (H): GO REGULATION OF T CELL RECEPTOR SIGNALING PATHWAY; (I): GO INTERFERON GAMMA MEDIATED SIGNALING PATHWAY; (J): GO INTERLEUKIN 4 PRODUCTION; (K): GO INTERLEUKIN 1 BETA PRODUCTION; (L): GO REGULATION OF LYMPHOCYTE APOPTOTIC PROCESS; (M): GO CD4 POSITIVE ALPHA BETA T CELL ACTIVATION; (N): GO RESPONSE TO INTERFERON GAMMA; (O): GO REGULATION OF T CELL DIFFERENTIATION; (P): GO INTERFERON GAMMA PRODUCTION; (Q): GO B CELL MEDIATED IMMUNITY; (R): GO INTERLEUKIN 10 PRODUCTION; (S): GO REGULATION OF T HELPER CELL DIFFERENTIATION; (T): GO POSITIVE REGULATION OF CELL CELL ADHESION. Notes: FDR, false discovery rate; NES: normalized enrichment score; ES: enrichment score.

In order to learn more about the molecular mechanism differences between high- and low-UXT-AS1 expression groups, we compared this two phenotypes by GSEA, so as to avoid the defect that most of genes were not included in functional enrichment analysis because they did not meet the DEG standard. GSEA analysis with c2 reference gene set (c2.all.v7.0.symbols.gmt) indicated that low UXT-AS1 expression phenotype was significantly enriched in the following signaling pathways: PI3KCI pathway, CD40 signaling UP, interleukine 12 (IL12)-signal transducer and activator of transcription 4 (STAT4) pathway, 41BB pathway, IL27 pathway, INTEGRIN2 pathway, METASTASIS, IL12 pathway, tumor necrosis factor receptor 2 (TNFR2) pathway, T cell receptor signaling pathway, cytotoxic T-lymphocyte associated protein 4 (CTLA4) pathway, B cell receptor signaling pathway, CD8-TCR pathway, programmed cell death 1 (PD1) signaling, TOB1 pathway, IL4 signaling, TP53 targets, natural killer cell mediated cytotoxicity, TNF receptor superfamily TNFSF members mediating non canonical NF-KB pathway, cytokine cytokine receptor interaction (**Figure 17A-T** and**Table S11**). GSEA analysis with c5 reference gene set (c5.all.v7.0.symbols.gmt) indicated that low UXT-AS1 expression phenotype was significantly enriched in tumor necrosis factor superfamily cytokine production, regulation of B cell receptor signaling pathway, B cell proliferation, T cell differentiation, lymphocyte differentiation, T cell proliferation, activation of Janus Kinase activity, regulation of T cell receptor signaling pathway, interferon gamma mediated signaling pathway, interleukin 4 production, interleukin 1 beta production, regulation of lymphocyte apoptotic process, regulation of T cell differentiation, response to interferon gamma, interferon gamma production, CD4 positive alpha beta T cell activation, B cell mediated immunity, interleukin 10 production, regulation of T helper cell differentiation and positive regulation of cell-cell adhesion (**Figure 18A-T** and**Table S12**). Through GSEA analysis, we found that immune-related signaling pathways and biological processes were significantly enriched between high- and low-UXT-AS1 expression groups, and we speculated that UXT-AS1 might be involved in the regulation of tumor immune-related biological processes and signaling pathways in PDAC. It is worth noting that our results significantly enriched an important signaling pathway that related to tumor immunotherapy:PD1 signaling pathway. Therefore, we speculate that UXT-AS1 may be a potential important target for PDAC immunotherapy.

## Discussion

Previous studies have shown that UXT-AS1 is significantly up-regulation in CRC tumor tissues and is related to poor prognosis. The results of our study are consistent with previous results, which observed that UXT-AS1 was significantly up-regulated in PC, and the prognosis of PDAC patients with high UXT-AS1 expression was poor. The present study is the first time report the clinical significance of UXT-AS1 in PDAC.

We further explored the functional mechanism of UXT-AS1 by screening the UXT-AS1 co-expressed genes and the DEGs of different UXT-AS1 expression phenotypes, as well as GSEA approach. The enrichment analysis in this study suggested that UXT-AS1 may play a part in PC by participating in the regulation of immune-related biological processes, that is, all three enrichment analyses were enriched in B cell or T cell receptor signaling pathway. B cell receptor signaling pathway in PDAC can regulate tumor microenvironment and then participate in the regulation of tumor growth and anti-tumor immune response 20. T cell receptor signaling pathways also play a part in PC immunotherapy. Cui et al. found that the T cell receptor β repertoire of tumor tissues in different regions of PC is homogeneous and speculate that the cellular adaptive immune response is homogeneous, which can provide a basis for immunotherapy of PC 21. Co-expression genes of UXT-AS1 and GSEA analysis also enriched in IL12, IL4, natural killer cell mediated cytotoxicity and NF-KB signaling pathways. Study have shown that IL4 combined with lipopolysaccharide (LPS) stimulation can increase the secretion of inflammatory cytokines and change the cell migration rate of PC induced by macrophages 22. The previous review summarized that IL12 is a key factor in cellular immunity, which can be induced by promoting natural killer cells and T cells. It can also play an anti-angiogenesis function through IFN-gamma-inducible genes and by lymphocyte-endothelial cell cross-tall. The immunomodulatory and anti-angiogenic functions of IL12 make it a promising potential anticancer drug 23. IL12 has been shown to have an excellent antitumor effect in PC 24-26. Previous reviews have shown that NF-KB signaling pathway is associated with cell proliferation, invasion, anti-apoptosis, inflammation, angiogenesis, and chemotherapy resistance in PC 27. Moreover, NF-KB signaling pathway is also significantly related to chemotherapy sensitivity and survival of PC, and can serve as a drug target of PC targeted therapy 28-32. A systematic review report summed up a lot of evidence that NK cells play a critical role in PDAC and their potential therapeutic impact 33. Current research finds that duodenopancreatectomy and gemcitabine can cause an increase of NK cells in PC, thereby exerting anti-cancer effects, then improving the survival of PDAC patients 34, 35. Another review report concludes that NK cells have the ability to select and differentiate cancer stem cells in PC to induce changes the tumor microenvironment that inhibit tumor growth and metastasis. It is suggested that it can be used as an important means of immunotherapy for PC 36. By co-culture NK cells with PC cells, Sun et al. found that NK cells could inhibit the proliferation, migration and invasion of co-cultured PC cells. This study suggested that targeting tumor microenvironment and related molecular cross-talk could be used as a new strategy for PC immunotherapy 37. PDAC-derived extracellular vesicles can be attenuated NK cell cytotoxicity of PC stem cells, and further affect the metastasis ability of PC cells 38.

Biological processes and signal pathways such as regulation of immune response, DNA repair, regulation of cell adhesion, stem cell population maintenance, mitotic sister chromatid cohesion, regulation of cell proliferation, positive regulation of tumor necrosis factor production, positive regulation of T cell proliferation and cell surface receptor signaling pathway, which enriched by UXT-AS1 co-expressed genes, are mainly involved in basic cell states regulation and are closely related to tumor immunology. For the results of GSEA analysis, we have also discovered multiple tumor immune-related biological mechanisms that are not enriched in the UXT-AS1 co-expressed genes function enrichment analysis. Study shows that IL27 exerts anti-tumor effects in PC cells by inducing cell cycle arrest and apoptosis 39. Yao et al. revealed that IL27 can inhibit PC cell proliferation, migration, and invasion through M2 polarized tumor associated macrophages, while enhancing the drug sensitivity of gemcitabine in PC cells 40. As we all know, PD1 is an important immunosuppressive molecule, antibodies against the PD1/ PD-L1 axis have been shown to be an effective immunooncology strategy, including PC 41. Previous studies have shown that PD1 can become an important target for immunotherapy of PC by changing the tumor immune microenvironment 42, 43, and PD1 is closely related to epithelial-mesenchymal transition-related proteins in PC 44. PD1 combined with other immune-related indicators can be used as markers for diagnosis and prognosis of PDAC 45. Bai et al. founded that TOB1 was down-regulation in PC tumor tissues, and play a tumor suppressor gene role in PC 46. Zhou et al. used the RNA-seq for CD73 knockdown PC cell lines founded that TNFR2 was involved in 5'-nucleotidase ecto (NT5E, also known as CD73)-induced AKT/ERK signaling pathway activation of PC 47. TP53 is one of the driving genes of PC and can serve as a gene target for PC targeted therapy 48, 49, and wild-type p53 serve as a tumor suppressor gene in PC 50. The immunohistochemical expression level of p53 is significantly correlated with the prognosis of PC, and it can be used to identify PC patients with high hematogenous metastasis risk 51. Studies revealed that CTLA4 is a new immunotherapeutic target, and inhibition of CTLA4 can promote the activation of T cells and then play an anti-cancer role. The anti-cancer effect of CTLA4 in PC can be played through regulate the infiltration of CD4+ T cells , and regulate CD8+ T cells in CD25-expressing Th17 cells 52, 53. Sandin et al. found that local inhibition of CTLA4 in PC can play an anti-tumor effect 54. Interferon gamma can play an anti-cancer role in pancreatic cancer by mediating the CXCL8-CXCR2 axis and CD8 + T-lymphocytes to participate in the regulation of the PD1 signaling pathway 55, 56. In addition, the study of A et al. also revealed that interferon r can also inhibit cxcl8 through RhoGDI2/Rac1/NF-KB signaling pathway to regulate the proliferation and migration of pancreatic cancer cells 57. A study by Halma and his coworkers found that polymorphism of interferon gamma were closely associated with PC clinical outcome 58. The janus kinase/signal transducer and activator of tran-ions (JAK/STAT) signaling pathway can be involved in regulating cell proliferation, angiogenesis, and malignant phenotype in PC. It is also closely related to the tumorigenesis and progression of PC 59. The proliferation, invasion, and metastasis of PC cells can be regulated by targeting the AK/STAT signaling pathway 60-64, and also can be used to improve the efficacy of chemotherapy drugs in PC 65, 66. Moreover, study have shown that chemotherapeutic drugs can improve the anti-tumor effect in PC by up-regulating the expression of PD1, and its mechanism involves regulating the JAK/STAT signaling pathway 67. MiRNA targeted regulation of JAK/STAT signaling pathway can also suppress the malignant phenotype of PC 68. Previous studies have shown that CD40 signaling pathway can participate in the regulation of tumor immune microenvironment in pancreatic cancer, and thus play an anticancer role, which is a new immunotherapy strategy 69, 70. Targeting the regulation of CD40 by miRNAs can significantly affect the invasion and metastasis ability of PDAC, which is significantly related to the progress of PDAC 71. HE et al. showed that CD40 was abnormal in PC tumor tissues, and the high CD40 expression was notably correlated with TNM staging and lymph node metastasis of PC 72. Radiotherapy combined with anti-CD40 can mobilize T cells in the body and improve the immune status of PC, the strategy can be used to improve the progression and prognosis of PC 73-75. Agonist CD40 combined with chemotherapy drugs can significantly inhibit PC, and CD40 ligand can be used to predict the treatment effect in PC patients after chemotherapy 76, 77. In addition, Chung et al. demonstrated that the CD40 ligand can also be serve as a biomarker in PDAC diagnosis and prognosis 78. Changes of CD40 agonist antibody to tumor microenvironment can improve the blocking response to PD-L1 in PC mouse model 79. By reviewing the above literature, among the potential mechanisms of UXT-AS1 enriched in the present study, we found that most of the mechanisms involved in tumor immune regulation, indicating that UXT-AS1 may play a part in PC by take part in the regulation of tumor immune microenvironment. Therefore, it is speculated that UXT-AS1 may play a certain part in the immunotherapy of PC and may be a new strategy of PC immunotherapy.

In this study, we used whole-genome RNA-seq dataset of PDAC patients' tumor tissues between different UXT-AS1 expression groups to perform CMap analysis to screen and predict small molecule drugs that might target UXT-AS1. We obtained two small-molecule drugs that may target UXT-AS1 in PDAC. Quipazine is a potent 5-hydroxytryptamine agonist in peripheral organs, however, its function in cancers has been rarely reported before. This study is the first to reveal its potential as a UXT-AS1 targeting drug in cancers. For another drug, terazosin, previous studies have shown an anti-cancer effect in urinary tract tumors, especially in prostate cancer 80-86. Previous studies have shown that terazosin inhibits tumors in prostate cancer by inducing apoptosis 81, 84, 85, this mechanism may be played through a p53 and Rb independent pathway 82. In addition, Chang et al. showed that A also had anti-cancer effects on metastatic prostate cancer 80. Tahmatzopoulos et al. revealed that terazosin can reduce the distribution of tumor vascularity in transitional cell carcinoma and induce the apoptosis of bladder transitional cells 83. By reviewing the literature, we found that the role of terazosin in anti-cancer has been previously reported. Therefore, the two drugs screened in this study may also have anticancer potential in PDAC. In the drug-gene interaction network, we found that two DEGs may be the downstream target genes of the two drugs to target UXT-AS1 in PDAC. By reviewing previous studies, we found that there were fewer reports between SLC6A2 and tumors, but we found that CNR2 plays a role in multiple cancers. Previous studies have shown that CNR2 is significantly up-regulated in a variety of tumor tissues, including melanoma 87, gliomas 88, glioblastoma 89, hepatocellular carcinoma (HCC) 90 and non-small-cell lung cancer (NSCLC) 91. Functional studies suggest that CNR2 plays an oncogenic role in human epidermal growth factor receptor-2 (HER2)-positive breast cancer (BC) patients 92 and NSCLC 91. In addition, Perez-Gomez et al. also observed that prognosis of high CNR2 expression BC patients with HER2-positive is poor 92. However, opposite trend of survival analysis also can be observed, that is, high CNR2 expression patients had a favourable prognosis in HCC 90 and NSCLC 91. Functional studies also suggest that CNR2 plays a role as a tumor suppressor gene in endometrial carcinoma 93. Based on the above understanding, we speculate that quipazine and terazosin may participate in the targeting of UXT-AS1 in PDAC by regulating the downstream CNR2, thereby exerting anti-cancer effects. The quipazine also play a role for target the UXT-AS1 in PDAC by regulating SLC6A2.

Our study also has some limitations. This study is a single-cohort study and the sample size is not large enough, therefore, our results still required validated in large cohorts. This study mainly explored the biological functional mechanisms of UXT-AS1 through bioinformatics analysis tools, the results still needs to be verified by *in vivo* and* in vitro* experiment. Nevertheless, our study is the first time that reveals the prognostic value of UXT-AS1 in PDAC, and to comprehensively analyze its biological functional mechanisms in PDAC through a variety of bioinformatics analysis tools based on genome-wide RNA-seq data sets. Through our current study, we preliminarily elucidated the biological functional mechanisms of UXT-AS1 in PDAC, which can provide a basis for future study on UXT-AS1. In this study, various physiological and pathological changes induced by UXT-AS1 in early PDAC were obtained at the genome-wide level through the TCGA genome-wide data set, and the biological mechanism changes related to early PDAC were comprehensively discovered. Based on this, we found that UXT-AS1 can serve as a new prognostic biomarker for PDAC, moreover, two potential UXT-AS1 targeted drugs in PDAC were screened out, thereby helping to improve the efficacy of PDAC treatment.

## Conclusions

In this study, we successfully identified a novel prognostic lncRNA, which named UXT-AS1, by TCGA PDAC cohort, which can be used to predict overall survival after pancreaticoduodenectomy in PDAC patients. We also found that UXT-AS1 were markerly up-regulation in PDAC tumor tissues, which may be involved in PDAC tumorigenesis, and may have a potential to be a diagnostic biomarker. At the same time, the biological functional mechanisms of UXT-AS1 were also explored by using genome-wide RNA-seq data sets. We found that UXT-AS1 may involve in the regulation of the nuclear factor kappa beta, regulation of tumor necrosis factor, cell adhesion, T cell receptor signaling pathway, and numerous immune-related biological processes and signaling pathways. We also found two drugs targeting UXT-AS1 using CMap, which named quipazine and terazosin. Due to the limitations of this study, our results still need further exploration and verification.

## Supplementary Material

Supplementary figure S1.

Supplementary tables.
