# Supplementary material for: UXT antisense RNA 1 sever as a novel prognostic long non-coding RNA in early stage pancreatic ductal adenocarcinoma patients after receiving pancreaticoduodenectomy
Source: J Cancer. 2021 Feb 21;12(7):2122–39. doi: 10.7150/jca.46084 (PMC7974525; doi:10.7150/jca.46084)
Supplement: Supplementary file 1 — Supplementary figure S1. [file jcav12p2122s1.pdf]

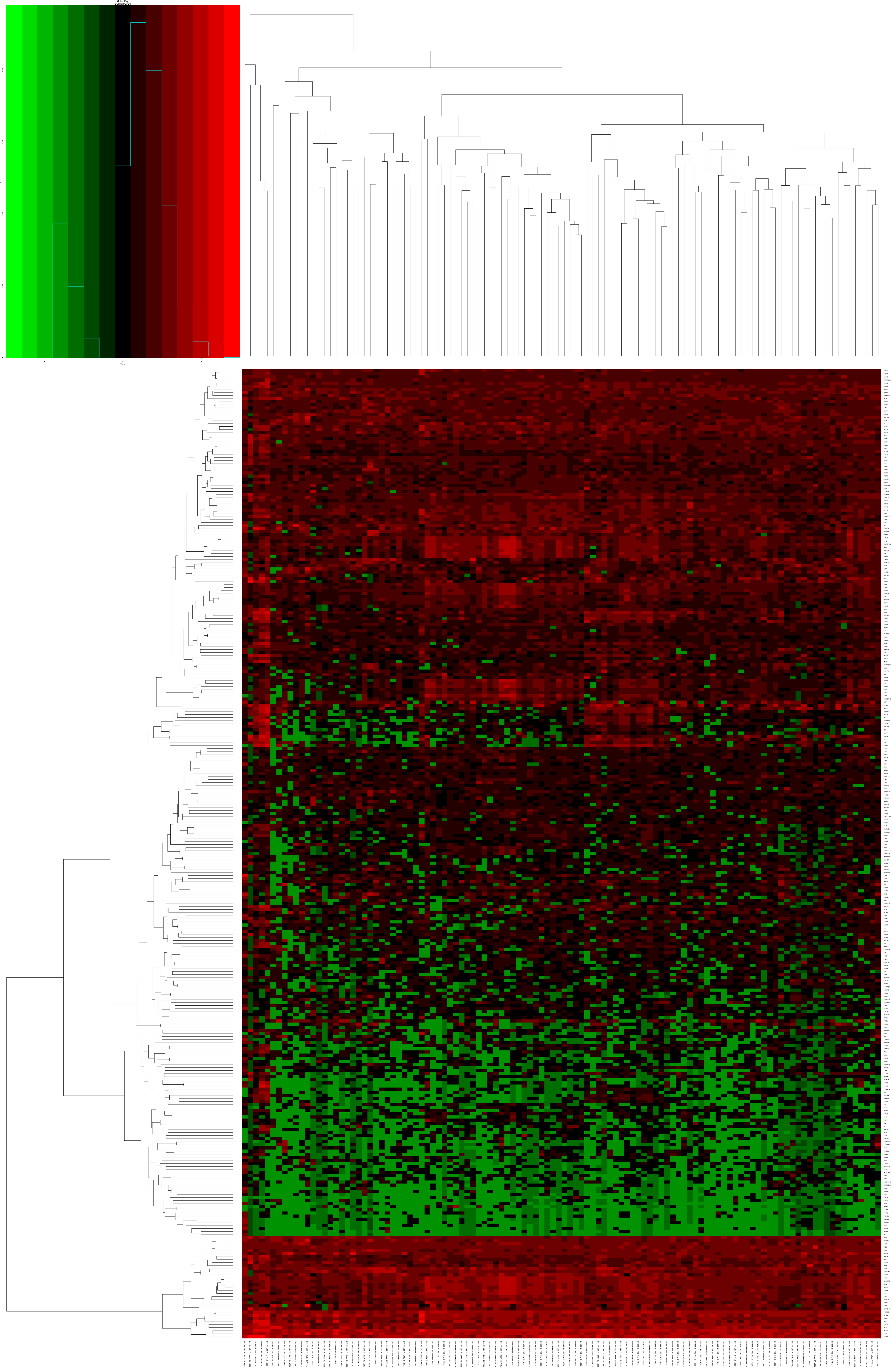

Figure S1. Heatmap of differentially expressed genes between high- and low-UXT-AS1 expression groups.
